# Enterovirus 71 VP1 Activates Calmodulin-Dependent Protein Kinase II and Results in the Rearrangement of Vimentin in Human Astrocyte Cells

**DOI:** 10.1371/journal.pone.0073900

**Published:** 2013-09-20

**Authors:** Cong Haolong, Ning Du, Tian Hongchao, Yang Yang, Zhang Wei, Zhang Hua, Zhang Wenliang, Song Lei, Tien Po

**Affiliations:** 1 Center for Molecular Virology, CAS Key Laboratory of Pathogenic Microbiology and Immunology, Institute of Microbiology, Chinese Academy of Sciences, Beijing, P. R. China; 2 Graduate School of the Chinese Academy of Sciences, Beijing, P. R. China; University of Birmingham, United Kingdom

## Abstract

Enterovirus 71 (EV71) is one of the main causative agents of foot, hand and mouth disease. Its infection usually causes severe central nervous system diseases and complications in infected infants and young children. In the present study, we demonstrated that EV71 infection caused the rearrangement of vimentin in human astrocytoma cells. The rearranged vimentin, together with various EV71 components, formed aggresomes-like structures in the perinuclear region. Electron microscopy and viral RNA labeling indicated that the aggresomes were virus replication sites since most of the EV71 particles and the newly synthesized viral RNA were concentrated here. Further analysis revealed that the vimentin in the virus factories was serine-82 phosphorylated. More importantly, EV71 VP1 protein is responsible for the activation of calmodulin-dependent protein kinase II (CaMK-II) which phosphorylated the N-terminal domain of vimentin on serine 82. Phosphorylation of vimentin and the formation of aggresomes were required for the replication of EV71 since the latter was decreased markedly after phosphorylation was blocked by KN93, a CaMK-II inhibitor. Thus, as one of the consequences of CaMK-II activation, vimentin phosphorylation and rearrangement may support virus replication by playing a structural role for the formation of the replication factories. Collectively, this study identified the replication centers of EV71 in human astrocyte cells. This may help us understand the replication mechanism and pathogenesis of EV71 in human.

## Introduction

Enterovirus 71 (EV71) is a single-stranded RNA icosahedral virus 30 nm in diameter belonging to the genus Enterovirus within the Picornaviridae family. In young children, its infection usually causes hand, foot and mouth disease (HFMD) which is characterized by several days of fever and vomiting, ulcerative lesions in the oral mucosa and vesicles on the backs of the hands and feet [1]. EV71 infections are usually accompanied by severe neurological complications such as aseptic meningitis, acute flaccid paralysis, encephalitis and other rarer manifestations [2], [3]. These neurological complications can sometimes be fatal and neurogenic pulmonary edema is thought to be the main disease process in fatal cases. It has also been postulated that overwhelming virus replication, combining with the induction of toxic inflammatory cytokines and cellular immunity resulting from tissue damage, are possibly the process of pathogenesis [4], [5]. Although the initial viral illness often is self-limited, EV71 infection may result in long term neurologic and psychiatric effects on the central nervous system (CNS) in children. Enterovirus 71 infection involving the CNS and cardiopulmonary failure may be associated with neurologic sequelae, delayed neurodevelopment and reduced cognitive functioning [6]. However, available treatments for EV71 infection and HFMD are limited as there is currently no effective chemoprophylaxis or vaccination for HFMD or EV71 infection.

Members of the Picornaviridae have similar particle morphology and genome organization, but several studies have revealed important differences in the replication of picornaviruses from different genera [7]. Picornavirus infections usually result in the formation of membranous structures in infected cells, many of which involve complex membrane rearrangements. Poliovirus (PV), enterovirus 11 (EV11) and encephalomyocarditis virus (EMCV) infections induce heterogeneously sized vesicles arranged as tightly packed clusters, while the vesicles in human parechovirus-1 (HpeV-1) infected cells are homogeneously sized, less numerous and do not associate to form tight clusters. Thus, the membrane vesicles induced by picornaviruses from different genera are different. Many studies suggest that the vesicular structures in infected cells are the virus factories. For example, Coxsackievirus B3 (CV-B3) infection induces autophagosome-like structures to serve as membrane scaffolds which support virus replication [8]. PV infection induces vesicles in a rosette-like arrangement around the replication complex. To date, the mechanism by which these vesicles are generated is still unknown. EV71 infection has been shown to induce the formation of autophagosome-like structures which is beneficial for virus replication [9]. However, little is known about the membrane rearrangement or the development of a specialized area for virus replication in EV71 infected cells.

Vimentin is a type III intermediate filament that play important roles during virus infections, including the recruitment of viral proteins or genomes, prevention of the movement of viral components into the cytoplasm, concentration of structural proteins at sites of assembly and providing a scaffold for virus assembly [10], [11]. As many virus infections are accompanied by a rearrangement and even a loss of cellular filaments, especially vimentin and actin, we have investigated potential changes in vimentin intermediate filaments and actin filaments during EV71 infection. The rearrangement of vimentin usually involves the phosphorylation of serine residues at the N terminal domain [12]–[14]. Such phosphorylation is believed to reduce interactions of the N terminal domains between the vimentin filaments and facilitates the disassembly or redistribution of filaments. In the present study, we investigated the involvement of vimentin in EV71 replication and the effects of virus infection on vimentin structure. We showed that EV71 infection activated CaMK-II, resulting in the phosphorylation of vimentin at serine 82. The serine 82 phosphorylated vimentin was rearranged, leading to the formation of virus replication centers.

## Materials and Methods

### Reagents and antibodies

N-decyl-b-D-maltopyranoside (DDM) was obtained from Affymetrix, CA, USA. 2-(4- Amidinophenyl)-6–indolecarba-midine dihydrochloride (DAPI) staining solution, Mito-tracker, KN93, Nocodazol and the protease-inhibitor cocktail were all obtained from Sigma Aldrich, MO, USA. Anti-EV71 monoclonal antibody was obtained from Millipore, MA, USA. Mouse Anti-BrUTP was obtained from R&D Systems, MN, USA. FuGENE transfection reagent was obtained from Roche, IN, USA. Rabbit anti-vimentin polyclonal antibody, mouse anti-β actin monoclonal antibody, mouse anti-β tubulin monoclonal antibody, mouse anti-α tubulin monoclonal antibody, rhodamine (TRITC)-conjugated anti-rabbit IgG antibody, FITC conjugated anti-rabbit IgG antibody, FITC conjugated anti-mouse IgG, HRP conjugated anti-mouse IgG and HRP conjugated anti-rabbit IgG were all obtained from Santa Cruz Biotechnology, CA, USA. Mouse anti-phospho vimentin (ser 55) was obtained from Abcam, MA, USA. BSA was obtained from BD, MA, USA. Rabbit anti-phospho vimentin (ser 82) monoclonal antibody, rabbit anti-phospho vimentin (ser 77) monoclonal antibody, rabbit anti-phospho vimentin (ser 38) monoclonal antibody, rabbit anti-CaMK-II monoclonal antibody and rabbit anti-CaMK-II (ser 286) monoclonal antibody were all obtained from Epitomics, CA, USA. Rabbit anti-PKA, anti-PKC and anti-CDK1 polyclonal antibodies were all purchased from Bioss, MA, USA.

### Cell cultures and virus strains

The prototype Enterovirus 71 (EV71) BrCr strain was a gift from Prof Qi Jin (Institute of Pathogen Biology, Chinese Academy of Medical Sciences, Beijing, PR China) [15]. The human astrocytoma cell line U251 and RD human rhabdomyosarcoma cells were propagated and maintained in Double Modified Eagle's Medium (DMEM) supplemented with antibiotics (penicillin and streptomycin) and 10% fetal bovine serum (Invitrogen, CA, USA) at 37°C in the presence of 5% CO_2_ [16], [17].

### Preparation of vimentin knockdown cell lines

In order to investigate potential roles for vimentin in EV71 infection, a vimentin knockdown cell line (VK-U251) was constructed using a retrovirus vector that stably expressed the siRNA specific to vimentin. To build VK-U251, a shRNA targeting the human vimentin gene (shVim) as reported previously [18] and a control shRNA (shControl) were designed. In order to facilitate the formation and processing of the shRNA, a loop sequence (TTCAAAGAGA) was designed in the middle area of all shRNAs. The sequences of the two shRNAs were: shVim, 5′-GAT CCG CTA TGT GAC CAC ATC CAC TTC AAG AGA GTG GAT GTG GTC ACA TAG CTT TTT TG-3′; and shControl, 5′-GAT CCC CAC CAT GCA CGT ATG TCA TTC AAG AGA TGA CAT ACG TGC ATG GTG GTT TTT TG-3′. The corresponding complementary oligonucleotides were also synthesized to produce DNA duplexes of each of the shRNAs. The shRNA oligonucleotides were annealed and ligated to the BamH I and EcoR I sites behind the human U6 promoter of pSIREN-RetroQ vector and the constructs confirmed by DNA sequencing. Phoenix cells were plated and transfected with the resultant pSIREN-RetroQ-siVim or pSIREN-RetroQ-siControl plasmid with a helper plasmid by using FuGENE transfection reagent. At 48 h post transfection, cell supernatants containing the recombinant retroviruses were harvested and used to infect U251 cells. The infected cells were screen by using puromycin at a concentration of 5 μg ml^−1^. Vimentin expression in VK-U251 and control cells was analyzed by western blot analysis.

### Virus propagation, purification and titer determination

Semi-confluent monolayers of RD cells were infected with approximately 2 PFU of EV71 per cell. After adsorption for 30 min at 37°C, the cells were washed twice with PBS buffer and overlaid with MEM containing 10% calf serum. The infected culture fluids were harvested daily from day 2 to day 6, and replaced with fresh MEM containing 10% calf serum after each harvest. Virus purification was done as described previously [19]. Briefly, after a low speed centrifugation, the culture fluids were passed through a 0.22 μm filter (Millipore, MA, USA). Viruses in the fluids were precipitated with 7% polyethylene glycol supplemented with 0.15 M NaCl and then purified by centrifugation through a 10 to 50% (w/w) linear sucrose gradient in a Beckman SW28 rotor for 12 h at 25000 g at 4°C. The virus pelleted from the gradient fractions was resuspended in TN buffer (0.1 M NaCl, 50 mM Tris, pH 7.4).

To determine virus titers, RD cells were plated into 96 well dishes, incubated overnight, and then infected with the serially diluted virus preparations. After adsorption for 30 min, the virus suspensions were replaced with MEM containing 2% fetal bovine serum. The cultures were incubated at 37°C for 5 days and plates that displayed cytopathic effects were counted. Virus titers were determined by the Reed-Muench method.

### Cell protein extraction and western blot

Cultured cells were collected and washed three times with PBS. After a short centrifugation at 800 g, the cell pellets were resuspended in cell lysis buffer (0.3% DDM in PBS) containing a protease inhibitor cocktail and incubated in ice for 30 min. After centrifugation at 12000 g for 15 min at 4°C, the soluble (supernatant) and insoluble (pellet) fractions were collected. Protein concentration of each sample was measured using the Bradford method (Bio-Rad Laboratories, CA, USA). Proteins (50 μg) were heat-denatured in sample-loading buffer (50 mM Tris-HCl, pH 6.8, 100 mM DTT, 2% SDS, 0.1% bromophenol blue and 10% glycerol), separated by SDS-polyacrylamide gel electrophoresis (12% polyacrylamide, 0.1% SDS) and electro-blotted to a polyvinylidene fluoride membrane. The membrane was blocked with 5% skim milk in TBST buffer (50 mM Tris-HCl, 100 mM NaCl and 0.1% Tween-20, pH 7.4) and incubated for 90 min at 37°C with the selected primary antibodies. The signals were visualized by a subsequent chemiluminescence reaction with the corresponding horseradish peroxidase conjugated IgG (1∶3000) in the ECL system (Invitrogen, CA, USA). Total vimentin in cells was analyzed by boiling cells with sample-loading buffer and subjected to immunoblot with antibody to vimentin.

### Immunofluorescence confocal microscopy

Cells were grown on cover slips and infected with EV71 or uninfected. Cell microtubules were depolymerized by incubating the cells in 3 μM Nocodazol for 2 h at 37°C and repolymerized by removal of the Nocodazol by washing the cells several times with DMEM. For CaMK-II inhibition, the cells were incubated with 40 μM KN93 for 16 h at 37°C. Cell mitochondria were stained using mito-tracker red (50 nM) according to the manufacturer's instructions. The cells were then fixed in paraformaldehyde in phosphate buffer (pH 7.4) for 30 min, and then permeabilized with 0.02% triton X-100 for 5 min. The cover slips were then rinsed with PBS and incubated with 5% BSA in PBS for 1h at room temperature to block any residual aldehyde groups. After 2 washes with PBS, the cells were incubated for 1 h with the selected antibodies. After washing thrice with PBS, the cells were incubated for 45 min with a FITC conjugated goat anti-rabbit or a TRITC conjugated goat anti-mouse IgG antibody. After washing thrice with PBS, cell nuclei were stained with DAPI. The cells were then examined under an inverted fluorescence or a confocal laser scanning microscope using an excitation wavelength of 568 nm or wavelengths of 490 nm and 355 nm.

### Transmission electron microscopy

Electron microscopy was performed as described previously [20]. Briefly, cell samples were washed three times with PBS, trypsinized and collected by centrifuging. The cell pellets were fixed with 4% paraformaldehyde overnight at 4°C, post-fixed with 1% OsO_4_ in cacodylate buffer for 1 h at room temperature and dehydrated stepwise with ethanol. The dehydrated pellets were rinsed with propylene oxide for 30 min at room temperature and then embedded in Spurr resin for sectioning. Images of thin sections were observed under a transmission electron microscope (JEM1230, Tokyo, Japan).

### Pull-down assay and co-immunoprecipitation

Pull-down assay was performed as described before [21]. For co-immunoprecipitation experiments, vimentin or EV71 compartments were immobilized on anti-vimentin or anti-Flag monoclonal antibody-conjugated agarose beads. After washing three times with PBS and once with lysis buffer, beads were incubated with U251 cell lysate overnight at 4°C with gentle rocking. After washing with lysis buffer, the incubated beads were boiled and proteins in the supernatant were collected and subjected to western blotting analysis.

### Fluorescence in situ hybridization

Fluorescence in situ hybridization (FISH) was performed as described previously [22]. In short, cells were fixed, permeabilized and quenched as described for immunofluorescence, using RNAse free solutions. The FITC or rhodamine-conjugated DNA probes complementary to the EV71 RNA genome were synthesized (5′–CCC AGT TGG CAT ACC CGT TCG GAT TGG TGG TGC CCT TTA GAG GAA GAT-3′ and 5′-GAT TTC GGC GGC TTG AAG TGC TGG TAC TTT TCC AGT GTC TAA GCG ATG AC-3′) and dissolved in water, and diluted 1∶10 in hybridization buffer (50% formamide, 10 mM Tris-HCl (pH 7.4), 600 mM NaCl, 10% dextran sulfate, 10 mM dithiothreitol, 0.05% bovine serum albumin, 0.1% sodium dodecyl sulfate (SDS), 200 μg salmon sperm DNA, and 100 μg yeast tRNA per ml) and hybridized to the cells at 40°C for 8 h. After four washes in 0.1% SSC (1% SSC is 150 mM NaCl and 15 mM sodium citrate) for 10 min each, cells were incubated with selected antibodies and subjected to immunofluorescence.

### RNA labeling and localization

Newly synthesized RNA was detected by bromouridine (BrU) incorporation. Twelve hours before virus infection, about 5×10^3^ U251 cells were plated per well in a 24-well plate. The following day, cells were infected with EV71 and then transfected with Bromo–UTP (BrUTP) using FuGENE transfection reagent according to the manufacturer's instructions. Briefly, for each well, 0.6 μl of FuGENE was added to 20 μl of OptiMEM (Invitrogen, CA, USA). After incubating for 10 min at room temperature, 1 μl of 10 mM BrUTP was added. After vortexing for one second to mix the contents, the transfection complex was incubated for 20 min at room temperature. The mixture was then added to the cells immediately. After swirling, the transfected cells were incubated indicated time at 37°C in 5% CO_2_. Cells were then fixed and subjected to immunofluorescence analysis using a mouse anti-BrdU antibody.

### Plasmid construction and protein expression

To create expression vectors expressing either EV71 VP1, VP2, VP3, 2C, 3D, 2A or 3C, EV71 genomic RNA was extracted from the supernatant of virus infected RD cells using a virus genome extraction kit. Single-stranded cDNA was then synthesized from the purified virus RNA by reverse transcription (RT) (Promega). Each of the VP1, VP2, VP3, 2C, 3D, 2A and 3C genes was amplified from the cDNA by PCR over 34 cycles of denaturation at 98°C for 10 sec, primer annealing at 55°C for 30 sec and extension at 72°C for 1.5 min, using VP1 sense primer CGC GGA TCC GAC AGA GTG GCA GAT GTG ATT G and anti-sense primer 5′- CCG GAA TTC TTA GAG CGT AGT GAT TGC CGT TC; VP2 sense primer 5′-CCC AAG CTT TCT CCC TCT GCT GAA GCA TGT GGC-3′ and anti-sense primer 5′-CCC AAG CTT TTA CTG CGT AAC TGC CTG CCT GAG AC-3′; VP3 sense primer 5′-CCC AAG CTT GGT TTC CCC ACT GAA TTG AA-3′ and anti-sense primer 5′-ACG CGT CGA CTT ATT GAA TAG TGG CCG TTT G C-3′; 2C sense primer 5′-CGC GGA TCC AGT GCC TCA TGG CTA AAG-3′ and anti-sense primer 5′-CCG GAA TTC TTA TTG AAA GAG TGC TTC TAT AGT ATT-3′; 3D sense primer 5′-CGC GGA TCC CCC AGC TTA GAC TTC GCC TTG TCT -3′ and anti-sense primer 5′-CCG GAA TTC TTA TTG CTC GCT GGC AAA ATA ACTCCT -3′; 2A sense primer 5′-CGC GGA TCC AAA TTC GGT CAG CAG TCT GGG GC -3′ and anti-sense primer 5′-CCG GAA TTC TTA CTG CTC CAT CGC TTC CTC ATC TAG -3′; 3C sense primer 5′-CGC GGA TCC CCC AGC TTA GAC TTC GCC TTG TCT -3′ and anti-sense primer 5′-CCG GAA TTC TTA TTG CTC GCT GGC AAA ATA ACT CCT -3′. Each of the PCR products was separated by electrophoresis on 1.0% agarose gels, gel purified and then cloned into the same restriction sites of the pCMV-Flag after being digested with Hind III and Sal I, or BamH I and EcoR I, respectively. To create expression vector expressing GFP-250 (GFP fused at its COOH terminus to a 250–amino acid fragment of the cytosolic protein p115) [23], the 250–amino acid fragment of the p115 was amplified from the cDNA of U251 cells by PCR, using sense primer 5′- CGG GAT CCA ATT TCC TCC GCG GGG TAA TGG -3′ and anti-sense primer 5′- CGG AAT TCC TAG ATA TGA TCT AGA TCC TTG CC. The PCR products was then cloned into the same restriction sites of the pcDNA 3.0-EGFP after being digested with BamH I and EcoR I. The recombinant plasmids were sequenced and then transfected separately into U251 cells using FuGENE transfection reagent according to the manufacturer's instructions. The expression of each protein was confirmed by western blot analysis.

## Results

### Involvement of vimentin in EV71 replication

**Figure 1:**
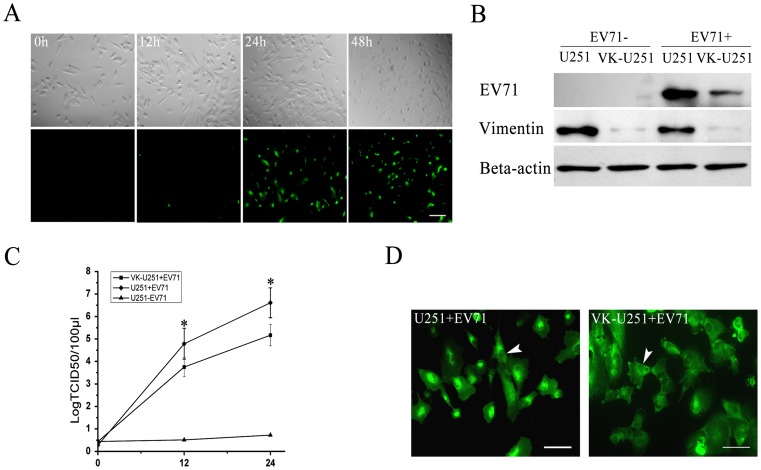
Analysis of EV71 infection and replication in U251 and VK-U251 cells. (**A**) Cytopathic effects of EV71 infection in human astrocytoma U251 cells. U251 cells were infected with EV71 at an MOI of 2 PFU cell^−1^. Cell morphology was observed at various times postinfection as indicated by light microscopy (top four panels). Virus infection was detected by immunofluorescence staining for virus VP1 protein (bottom four panels) using indirect immunofluorescence microscopy. Bar: 500 μm. (**B**) Detection of EV71 VP1, vimentin and actin (internal control) in EV71 infected U251 and VK-U251 cells by Western blot analysis. Each cell line was infected with EV71 (EV71+) using uninfected cells as controls (EV71−). They were harvested at 24 h postinfection and processed for western blot analysis using antibodies specific to EV71 VP1 (EV71), vimentin or actin as described in Materials and Methods. (**C**) Measurement of virus titers in the supernatants of EV71 infected U251 (U251 + EV71) and EV71 infected VK-U251 (VK-U251 + EV71) cells, using uninfected U251 cells as controls (U251 – EV71). Virus titration was performed at 0, 12 and 24 h postinfection. The data show the mean virus titers ± SD from three independent experiments. Asterisks indicate significant differences at p<0.05 compared to control. (**D**) Immunofluorescence microscopy analysis of the intracellular distribution of EV71 proteins in U251 and VK-U251 cells at 24 h postinfection. Cells were infected with EV71 and stained with antibody to EV71. Positive reaction appears as green florescence. White arrow head shows the nuclei. Bar: 100 μm.

We first examined whether EV71 could infect human astrocytoma cells. After infecting with EV71 at the indicated MOI of infection, U251 human astrocytoma cell morphology and virus replication were observed by using indirect immunofluorescence at increasing times postinfection. The results showed that EV71 could directly infect U251 human astrocytoma cells and numerous detached, round and floating cells displayed the signs of cytopathocity could be detected 48 hours postinfection (**Fig. 1A**).

Vimentin expression in the vimentin knockdown cell line (VK-U251) was compared to that of the normal U251 cells by Western blot analysis before infection with EV71. The results showed that there was little vimentin expression in the VK-U251 cells (**Fig. 1B**). Afer infection with EV71, virus replication decreased dramatically in the VK-U251 cells compared to U251 cells, as indicated by both a lower level of virus proteins in these cells (**Fig. 1B**) and a decrease in virus titers in the cell supernatants (**Fig. 1C**). In addition, the intracellular distribution of EV71 VP1 protein in VK-U251 cells was different from that in U251 cells. Most EV71 VP1 protein in infected U251 cells were localized near or around the nuclei, whereas in VK-U251 cells, they were dispersed throughout the cells and not concentrated in any specific region (**Fig. 1D**). Thus, the above results suggested that vimentin is important for EV71 replication in human astrocytoma cells and influenced the distribution of EV71 in these cells.

### Effect of EV71 infection on vimentin structure

**Figure 2:**
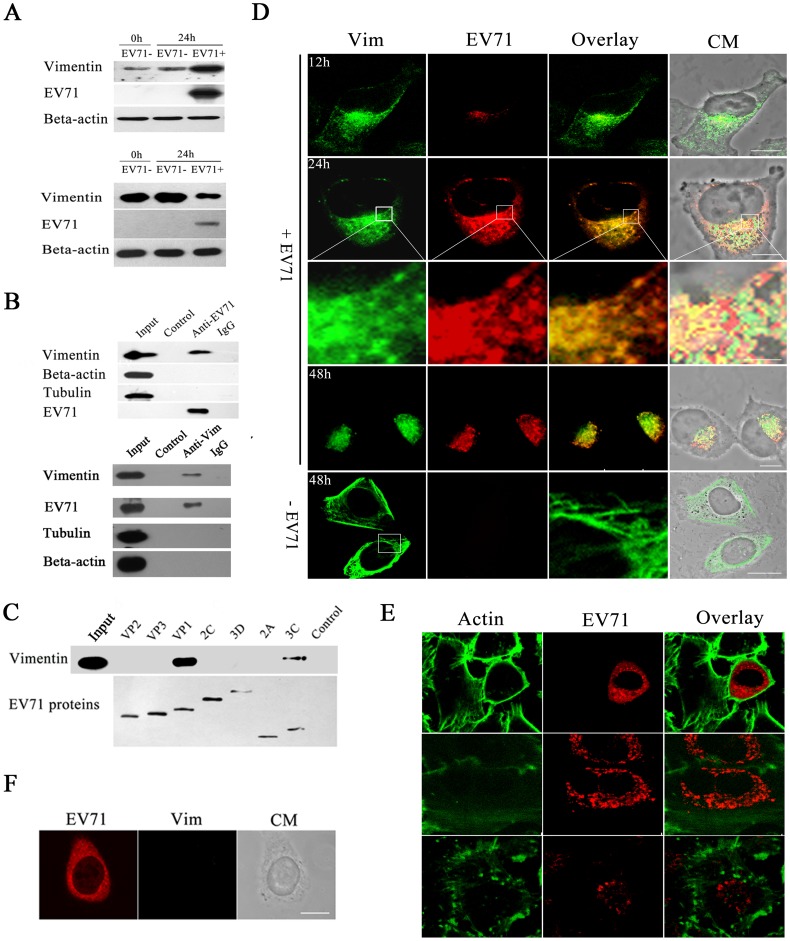
Detection and analysis of vimentin rearrangement caused by EV71 infection in human astrocytoma cells. (**A**) Western blot analysis of cell vimentin in the soluble (top panels) and insoluble (bottom panels) fractions of EV71 infected cells. Infected U251 cells (EV71+) were lysed at 24 h postinfection, as described in Material and Methods. Beta-actin was used as an internal control. Uninfected controls (EV71−) were similarly analysed at 0 and 24 h post infection. The figure shows the increase in production of soluble vimentin and concomitant reduction of insoluble vimentin in EV71 infected cells. (**B**) Analysis of the interactions between EV71 virus particles and cell vimentin, actin and tubulin by pull down assay (top panels) and co-immunoprecipitation (bottom panels) assays performed as described in Materials and Methods. The figure shows a western blot of the immunoprecipitated proteins. The lanes in the top panels are: lane input  =  cell lysate, lane control  =  agarose beads incubated with purified EV71 particles, lane anti-EV71  =  anti-EV71 monoclonal antibody-conjugated agarose beads incubated with purified EV71 particles, lane IgG  =  mouse IgG-conjugated agarose beads incubated with purified EV71 particles. The lanes in the bottom panels are: lane input  =  cell lysate, lane control  =  proteins from cell lysate incubated with IgG-conjugated agarose beads, lane anti-Vim  =  proteins from cell lysate incubated with vimentin monoclonal antibody-conjugated agarose beads. The results showed the specific binding of EV71 to vimentin and not to actin or tubulin. (**C**) Analysis of the interaction between vimentin and EV71 proteins. U251 cells were transfected with plasmids expressing each of the EV71 components as indicated on each lane. They were then lysed and immuno-precipitated with an antibody to the Flag tag and the immuno-precipitants were then analysed by western blot with antibodies specific to vimentin and Flag tag. The figure shows EV71 VP1 mediated the interaction between EV71 and vimentin. (**D**) Immunofluorescence analysis of the distribution of vimentin and EV71 in viral infected cells. U251 cells were infected with EV71 (+EV71) and fixed at the indicated time postinfection. Cells were then stained with antibodies to vimentin (Vim, green fluorescence) and EV71 (EV71, red florescence) and subjected to confocal microscopy analysis. An overlay of the vimentin and EV71 florescence is also shown (Overlay). Cell morphology (CM) was assessed by light microscopy. The figures showed cell vimentin (green) rearranged and reclustered to form aggresomes-like structures in the perinuclear region with EV71 proteins (red) with increasing time of EV71 infection. Bar: 20 μm; -EV71: mock infected controls. (**E**) Confocal immunofluorescence analysis of the distribution of actin and EV71 in viral infected cells. U251 cells were infected (+EV71) as described above. The actins in cells were depolymerized by treatment with actin polymerization inhibitor (Cytochalasin D; 1 μM) for 1h and repolymerized by the removal of cytochalasin D. The figures show no obviously changes in the integrity and distribution of cell actin (green)in viral infected cells (red) compared with mock infected cells. Bar: 20 μm. (**F**) Confocal immunofluorescence analysis of the distribution of vimentin and EV71 in viral infected VK-U251cells. The VK-U251 cells were infected with EV71 for 24 hours and then treated with antibodies to vimentin (Vim, green fluorescence) and EV71 (EV71, red florescence) as described in figure D. The figures show no aggresomes-like structures in the perinuclear region in infected VK-U251 cells. Bar: 20 μm.

Cells were infected with EV71 and the intracellular proteins were analysed by western blot using antibodies specific for actin and vimentin filaments. The results showed that at 24 h postinfection, the vimentin in the detergent soluble fractions had increased dramatically in EV71 infected cells when compared to uninfected cells (**Fig. 2A** **, top panel**). As expected, the vimentin in the insoluble fractions decreased correspondingly (**Fig. 2A** **, bottom panel**). In contrast, there were no obvious changes in actin levels in both the soluble and insoluble fractions after EV71 infection (**Fig. 2A**). Analysis of virus protein in these fractions showed that most of the VP1 protein was contained in the soluble fractions. There was also a small amount of VP1 in the insoluble fractions (**Fig. 2A** **, bottom panel**). Pull down assay and co-immunoprecipitation were performed to analyze the interactions of EV71 particles with microtubes, vimentin and actin. The result showed that EV71 particles could directly interact with vimentin, but not with actin or microtubes (**Fig. 2B**). Co-immunoprecipitation was performed to further determine which EV71 protein component contributed to the interaction. The result showed that vimentin specific bands could only be detected in the EV71 VP1 and 3C precipitants (**Fig. 2C**), suggesting that mainly EV71 VP1 and 3C mediated the interaction between the virus and vimentin.

To further investigate the interactions between EV71 and vimentin, confocal immunofluorescence microscopy was performed to examine the co-localization of EV71 virus protein and vimentin. The results showed that the distribution of vimentin was clearly changed in EV71 infected cells. At 12 h postinfection, at which time little EV71 particles could be detected (**Fig. 2D**), some of the vimentin began to disassemble, as shown by a decrease in the amount of vimentin filaments in these infected cells (**Fig. 2D**). At 24 h postinfection, most of the vimentin filaments were disassembled and rearranged to form aggresome structures near the perinuclear region (**Fig. 2D**). In contrast, the vimentin was arranged in a filamentous network reaching the cell surface in the corresponding uninfected cells (**Fig. 2D**). Further studies showed that almost all the EV71 VP1 co-localized well with the vimentin aggresomes and arranged into a ring-like structure near the perinuclear region (**Fig. 2D**). At 48 h postinfection, almost all the disassembled vimentin filaments was rearranged and co-localized with EV71 VP1 near the perinuclear region (**Fig. 2D**). Overexpression of misfolded proteins caused formation of aggresomes and usually accompanied with the rearrangement of the vimentin intermediate filament [23]. We investigated the redistribution of vimentin by expressing a protein chimera GFP-250 in U251 cells. Result showed that GFP-250 protein produced an intense perinuclear fluorescence signal in U251 cells. The vimentin was collapsed into ring around the aggregated GFP-250 in the perinuclear region of the cell occupied by the aggresome **(Fig. S1)**. We also examined whether the actin skeleton is involved in aggresomes or aggresomes clustering. The actin in cells were depolymerized by treatment with actin polymerization inhibitor (Cytochalasin D; 1 μM) for 1 h and repolymerized by the removal of cytochalasin D. The distribution of actin and EV71 VP1 was then analyzed by confocal immunofluorescence microscopy. The results showed that there was no co-localization of aggresomes and actin. The addition of Cytochalasin D completely abrogated the actin cytoskeleton. The aggresomes in EV71 infected cells remained aggregated near the perinuclear region in the presence of cytochalasin D, indicating that the actin skeleton is not involved in aggresomes or aggresomes clustering (**Fig. 2E**). EV71 replication could also be detected in VK-U251 cells, but no aggresomes were observed (**Fig. 2F**), further indicating that the formation of aggresomes required vimentin.

### Vimentin rearrangement and vimentin phosphorylation in EV71 infected cells

**Figure 3:**
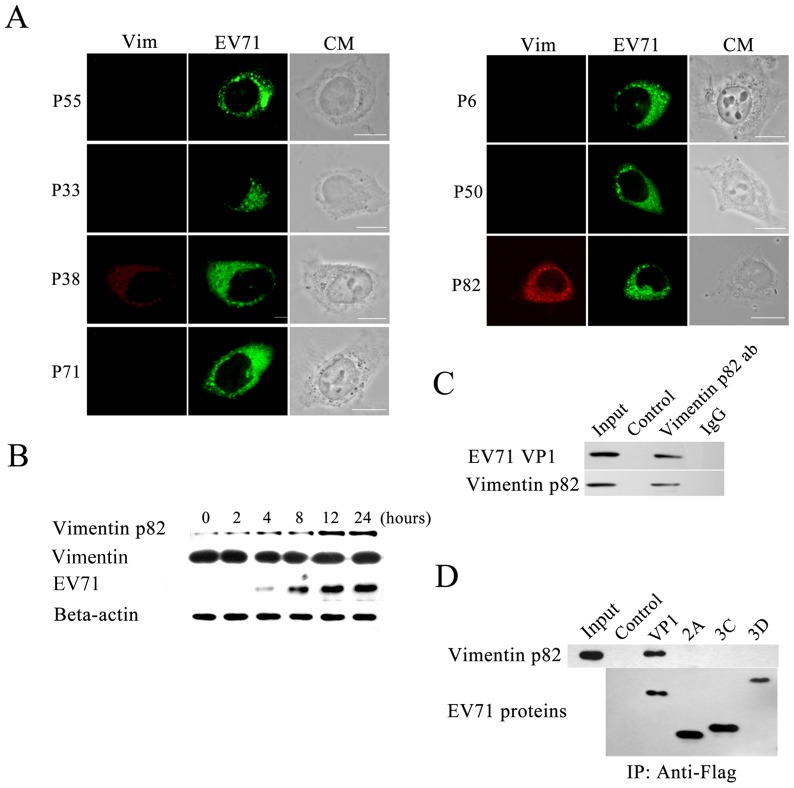
Analysis of vimentin phosphorylation involved in vimentin rearrangement during EV71 infection. (**A**) Confocal microscopy analysis of vimentin phosphorylation in U251 cells infected with EV71 showing only ser 82-p vimentin colocalized with aggresomes. At 24 h postinfection cells were stained with antibodies specific to either Ser-55-, Ser-33-, Ser-38-, Ser-82-, Ser-6-, Ser-50- or ser 71- phosphorylated vimentin (left column showing red florescence). EV71 was detected by immuno-staining using antibody against the EV71 VP1 (middle column showing green fluorescence). Bar: 20 μm. (**B**) Western blot analysis of EV71 infected U251 cells showing detection of increasing amounts of ser 82-p vimentin with time postinfection. Infected cells were harvested at the indicated hours postinfection, lysed and processed for immuno-blotting with antibodies specific to ser 82-p vimentin, vimentin, EV71 and Beta-actin (internal control). (**C**) Analysis of the interaction between EV71 and ser 82-p vimentin by immunoprecipitation showing the specific co-immunoprecipitation of EV71 and ser 82-p vimentin. Immunoprecipitation was performed as described in Materials and Methods followed by western blot of the precipitated proteins with antibodies to EV71 VP1 and ser 82-p vimentin. The lanes in the figure are: lane input  =  cell lysate, lane control  =  agarose beads with no treatment, lane vimentin p82 ab  =  agarose beads conjugated with ser 82-p vimentin antibody, lane IgG  = . agarose beads conjugated with mouse IgG. (**D**) Analysis of the interaction between EV71 VP1 and ser 82-p vimentin showing co-precipitation of EV71 VP1 and ser 82-p vimentin. U251 cells were transfected with plasmids expressing EV71 VP1, 2A, 3C, 3D. They were then lysed and immuno-precipitated with an antibody to the Flag tag and the immuno-precipitants were then immunoblotted with antibodies specific to ser 82-p vimentin and Flag tag.

Phosphorylation of the vimentin involved in aggresomes formation was investigated. U251 cells, fixed at 24 h postinfection, were examined by immunofluorescence microscopy using antibodies specific for phosphorylated vimentin filaments and EV71 antibodies. The results showed that serine 82 of vimentin was phosphorylated, and not ser-55, Ser-33, Ser-38, Ser-72, Ser-6 or Ser-50. Further, the phosphorylated vimentin was found to co-localize well with EV71 and formed aggresome structures in the perinuclear region (**Fig. 3A**). The phosphorylation of vimentin at ser 82 in infected cells was also examined by western blot. As shown in **figure 3B** **,** the levels of ser 82 phosphorylated vimentin (ser 82-p vimentin) increased concomintantly with the time of infection. To further determine if the ser 82-p vimentin could interact with EV71 proteins and forms the aggresomes, interaction between EV71 and ser 82-p vimentin was assessed by co-immunoprecipitation assays as described in Materials and Methods. The results showed that EV71 particles bound directly to ser 82-p vimentin (**Fig. 3C**). The interactions between ser 82-p vimentin and EV71 VP1, 2A, 3C, 3D were also analyzed by co-immunoprecipitation assays. Results showed that ser 82-p vimentin directly interact with EV71 VP1, but not 2A, 3C, 3D. This indicated that EV71 particles may bound to ser 82-p vimentin through VP1 (**Fig. 3D**).

### Association of the vimentin aggresomes with the site of EV71 replication

**Figure 4:**
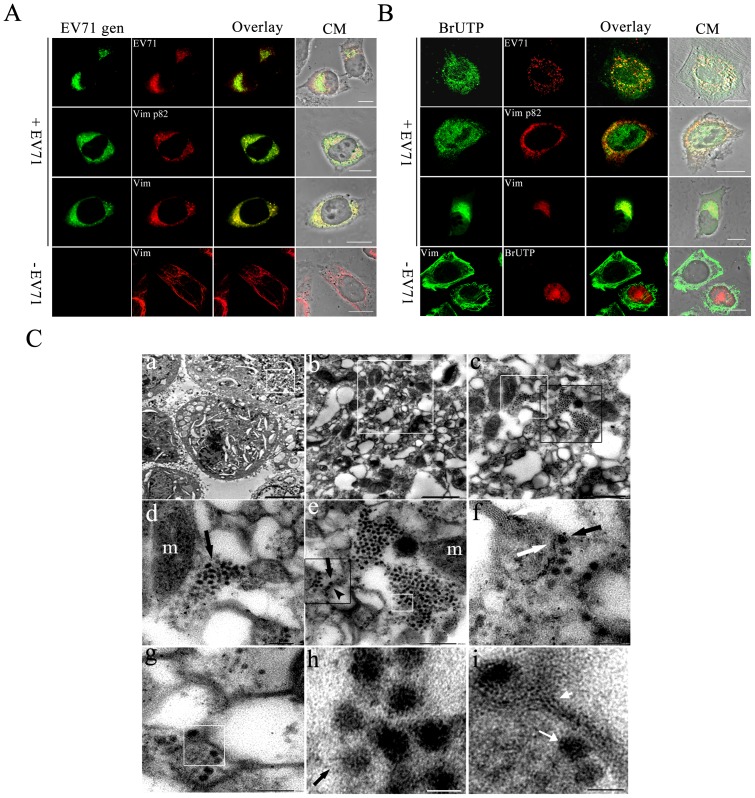
Analysis of the role of vimentin aggresomes in EV71 replication. (**A**) Detection of EV71 genome, vimentin and ser 82-p vimentin distribution in virus infected cells showing the co-localization of EV71 genome with EV71 VP1 in perinuclear region. U251 cells were infected with EV71 for 24 h. Cells were fixed, permeabilized, and quenched as described for immunofluorescence. Cells were then hybridized with a FITC-conjugated DNA probe complemented with EV71 VP1 gene (EV71 gen) as described in Material and Methods. Cells were then stained with antibodies to EV71 VP1 (EV71), vimentin (Vim) or ser 82-p vimentin (Vim p82) and subjected to immunofluorescence analysis. CM: Cell morphology. Bar: 20 μm. (**B**) A study of the distribution of newly synthesized viral RNA, vimentin and ser 82-p vimentin. Newly synthesized RNA in EV71 infected cells (+EV71) was labeled with BrUTP as described in Material and Methods. The incorporated BrU was stained by using a mouse anti-BrdU antibody (BrU; green). After washing with PBS, cells were stained with antibodies to EV71, ser 82-p vimentin (Vim p82) or vimentin (Vim). Newly synthesized RNA (red) and vimentin (green) in uninfected control (−EV71) was also showed. Bar: 20 μm. (**C**) Analysis of the aggresomes in EV71 infected cells by transmission EM. Cells were infected with EV71 and prepared for EM as described in Materials and Methods. Panel a: TEM showing a high electron density region near the perinuclear (white box) in EV71 infected cell. Bar: 20 μm. Panel b: A magnified view of the boxed section in panel a. Bar: 500 nm. Panel c: A magnified view of the boxed section in panel b, showing irregular aggresomes-like structures and mitochondria aggregated. Bar: 300 nm. Panel d: A magnified view of the white boxed section in panel c, showing the intact viral-like particles and the incomplete viral-like particles were found localized on the surface or aggregated very close to the aggresomes and near to the mitochondria. m  =  mitochondria. Bar: 150 nm. Panel e: A magnified image of the black boxed section in panel c. The image shows viral-like particles (long black arrow) combined with filament structures (black arrow head). Note the accumulation of virus-like particles (white arrow) in the replication site. Bar: 200 nm. Panel f: An enlargement of the black box area in image e. Note that virus-like particles interact with filament-like structures (white arrow) and arranged very regularly (black arrow). Bar: 100 nm. Panel g: Another magnified view of the viral replication site showing filament-like structures and viral-like particles. Bar: 100 nm. Panels h and i shows an enlargement of the white box in panel e and white box in panel g, respectively. Black arrow in panel h shows a virus-like particle attached to filament-like structure. Short white arrow head in panel i shows filament-like structures. Long white arrow shows a viral –like particle. Bar: 30 nm.

Fluorescence in situ hybridization (FISH) was performed to determine if the aggresomes also contained EV71 genome. The distribution of EV71 genomes in U251 cells infected with EV71 were analysed at 24 h postinfection using two FITC-conjugated probes that specifically recognized the EV71 VP1 gene (**Fig. 4A**). The cells were then treated with antibodies specific to either vimentin, ser 82-p vimentin or EV71 (**Fig. 4A**) and subjected to confocal microscopy analysis. The results showed that most of the EV71 genomes detected co-localized with the vimentin aggresomes and EV71 proteins, although some of them spread into the cytoplasm.

Since most of the EV71 proteins and genome were found associated with the aggresomes, these aggresomes may represent EV71 RNA replication centers. To obtain evidence for or against this, the incorporation of BrUTP into newly synthesized viral RNA in EV71 infected cells was studied using an anti-BrdU antibody. The virus structural proteins were also detected with EV71 antibody. As shown in **Fig. 4B**, at 2 h post transfection with BrU, the incorporation of BrU can be detected as fluorescence dots in the perinuclear region which co-localized well with the EV71 proteins (**Fig. 4B**). In addition, the newly incorporated BrU also co-localized with aggresomes and ser 82-p vimentin in the perinuclear region (**Fig. 4B**). All these results strongly suggested that the aggresomes containing both virus proteins and virus-specific RNA.

To further support the above conclusion, transmission electron microscopy (TEM) was performed at 24 h postinfection to analysis the distribution of virus particles in infected cells. As showed in **Fig. 4C**, **panel a,** EV71 infection induced many large membranous vesicles seen dispersed throughout the cytoplasm. On the contrary, EV71-like particles, of 30 nm in diameter, were restricted to the perinuclear region where a mass of irregular aggresomes-like structures and mitochondria aggregated (**Fig. 4C** **, panel b**). These aggresomes were found close together, heterogeneous in size and some were clustered in rosettes (**Fig. 4C** **, panel c**). Many mitochondria aggregated adjacent to these aggresomes. When these aggresomes were examined under medium resolutions, we found that many of the EV71-like particles or incomplete particles were found localized on the surface or aggregated very close to the aggresomes and, at the same time, very near to the mitochondria (**Fig. 4C** **, panel c and d**). This result is in accord with our finding that EV71 infection led to the recruitment of mitochondria to the virus replication centers. At high resolutions, some virus-like particles were seen adhering to the outer surface of the aggresomes which appeared at as higher electro-density structures (**Fig. 4C** **, panel f, h and i**). In addition, we also observed a direct interaction between the virus particles and cell filaments in the virus aggregated regions. Most importantly, these aggresomes, in contrast to the membranous vesicles mentioned above, did not possess any membranes but did have some skeleton-like features as indicated by the presence of filament-like structures (**Fig. 4C** **, panel i**).

### Activation of calcium calmodulin kinase II during EV71 infection

**Figure 5:**
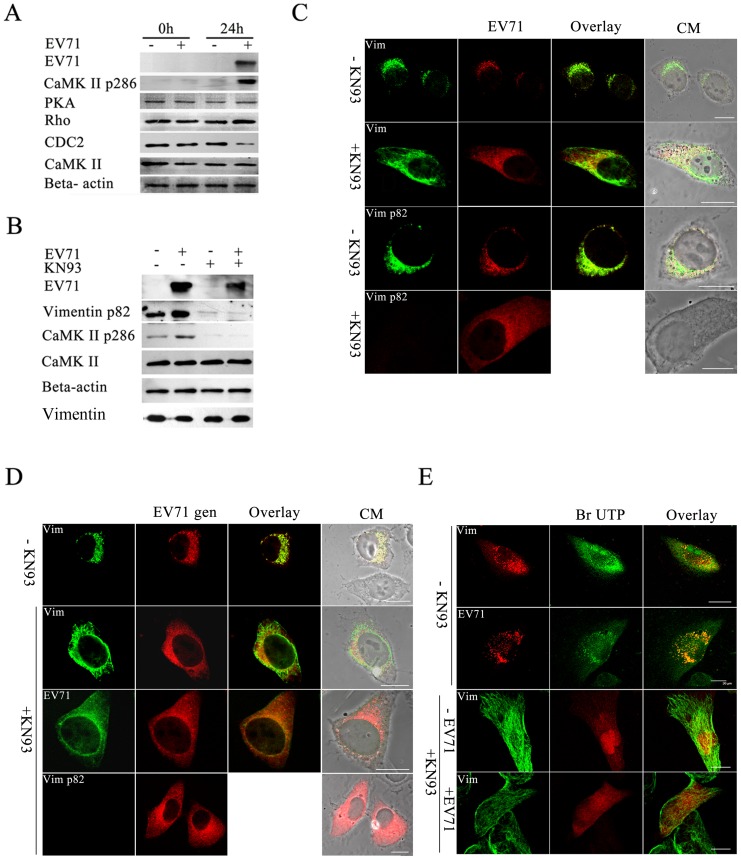
Analysis of CaMK-II activation and its roles in EV71 infection. (**A**) Western blot analysis of EV71 infected U251 cells showing the activation of CaMK-II during EV71 infection. Cell infected with EV71 (+) or mock infected (−) and incubated for 24 h (24 h) or unincubated (0h), respectively, were lysed and immunoblotted with antibodies against either PKA, CDC 2, Rho-kinase (Rho), CaMK-II, phosphorylated CaMK-II (CaMK-II p286), EV71 or Beta-actin internal control as shown on the left. The results showed that only an increase in the expression of CaMK-II p286 was detected after infection with EV71. (**B**) Western blot detection of CaMK-II p286, CaMK-II, EV71 VP1 and ser 82-p vimentin protein levels in cells treated with KN93. Infected (+ EV71) and uninfected U251 cells (− EV71) were incubated in the presence (+ KN93) or absence of KN93 (− KN93) and processed for western blot with antibodies against CaMK-II, phosphorylated CaMK-II (CaMK-II pThr 286), ser 82-p vimentin (Vimentin p82), EV71 and Beta-actin (internal control) as described in Materials and Methods. Total vimentin in cells was also analyzed. The antibodies used is labelled on the left. The figure shows the levels of CaMK-II Thr 286 phosphorylation, vimentin Ser-82 phosphorylation and EV71 proteins were decreased in the presence of KN93. (**C**) Immunofluorescence analysis of the distribution of vimentin (Vim), ser 82-p vimentin (Vim p82) and EV71 protein (EV71) in EV71 infected cells in the presence (+KN93) or absence of KN93 (− KN93) as described in Materials and Methods. The figure shows the fluorescence observed after treatment with the respective antibodies. The figure shows the aggresomes found in KN93 untreated cells disappeared after KN93 treatment. Bar: 20 μm. (**D**) FISH analysis of the effects of KN93 on vimentin aggresomes formation. U251 cells were infected with EV71 and treated (+ KN93) or untreated with KN93 (−KN93), and hybridized with DNA probe, followed by staining with antibodies to vimentin (Vim), ser 82-p vimentin (Vim p82) and EV71 (EV71), and subjected to confocal microscopy analysis as described in Materials and Methods. The figure shows the red fluorescence representative of the EV71 genome (EV71 gen) observed after DNA hybridization and the green fluorescence observed after blotting with the respective antibodyand an overlay of the two. CM: Cell morphology. Bar: 20 μm. (**E**) Analysis of the effect of KN93 on the distribution of newly synthesized viral RNA, vimentin and ser 82-p vimentin in viral infected cells. Cells were infected with EV71 and treated (+KN93) or untreated (−KN93) with KN93 as described above. The incorporated BrU to RNA was detected using a mouse anti-BrdU antibody (BrU) and is observed as fluorescence on the middle column. After washing, cells were stained with antibodies to EV71, vimentin (Vim), and serine 82-p vimentin (Vim p82) and the positive signals are observed as fluorescence on the left column.An overlay of the two are shown in the right column. CM: Cell morphology. Bar: 20 μm.

Since the phosphorylation of vimentin is regulated by many cell cycle control kinases such as Cdk1, PKC, Rho-kinase and CaMK-II [24]–[26] and that activated forms of CaM kinase II are responsible for phosphorylating vimentin at ser 82, EV71 infection might induce CaMK-II auto-phosphorylation at threonine 286, leading to an increase in the activity of CaMK-II. To confirm this, U251 cells infected with EV71 were collected at 24 h postinfection and subjected to western blot. The levels of activated forms of CaMK-II were detected by using antibody that specifically recognized threonine 286 of CaMK-II. The results showed that threonine 286-phosphorylated CaMK-II increased markedly in EV71 infected cells, while the total CaMK-II remained the same in both EV71 infected and uninfected cells. We also examined Cdk1, PKC and Rho-kinase levels in EV71 infected cells and the results showed that there was no obvious change in PKC and Rho-kinase levels compared to uninfected cells. However, the level of Cdk1 was decreased significantly (**Fig. 5A**).

To investigate the roles of ser 82-p vimentin in the EV71 replication sites, we examine the effect of inhibition of vimentin serine 82 phosphorylation on virus replication, by treating EV71 cells with KN93, a selective CaMK-II inhibitor which prevents the auto phosphorylation and activation of CaMK-II [27]. Western blot of KN93 treated cells (**Fig. 5B**) showed that the amounts of ser 82-p vimentin and activated forms of CaMK-II decreased dramatically after KN93 treatment, accompanied by a corresponding decrease in EV71 virus protein expression levels. To confirm that the presence of KN93 led to the inhibition of EV71 replication, virus titers in cells and supernatant was determined. The results showed that the number of infectious virus in KN93 treated cells and supernatant decreased significantly when compared to corresponding samples with no KN93 treatment **(Fig. S2)**.

Further analysis of the infected cells by immunofluorescence analysis showed that the aggresomes normally found in KN93 untreated cells (**Fig. 5C** **)** disappeared in the presence of KN93 (**Fig. 5C**), and most vimentin kept their original filamentous shape, similar to uninfected cells (**Fig. 5C**). Furthermore, **Fig. 5C** showed that in the presence of KN93, there was no immunofluorescence signal detected for ser 82-p vimentin indicating that KN93 prevented the phosphorylation of vimentin by CaMK-II. Analysis of virus distribution by immunofluorescence analysis showed that the small amount of EV71 virus produced in KN93 treated cells was detected throughout the cytoplasm instead of being assembled in the perinuclear region (**Fig. 5C**). The distribution of virus genome detected by FISH also showed that the EV71 genome or virus particles were dispersed throughout cytoplasm (**Fig. 5D**). RNA labeling also showed that accompanying with the disappearance of virus factories, the newly synthesized viral RNA was also dispersed in the cytoplasm (**Fig. 5E**). These results indicated that the phosphorylation of vimentin ser 82 was necessary for the formation of the virus replication centers.

**Figure 6:**
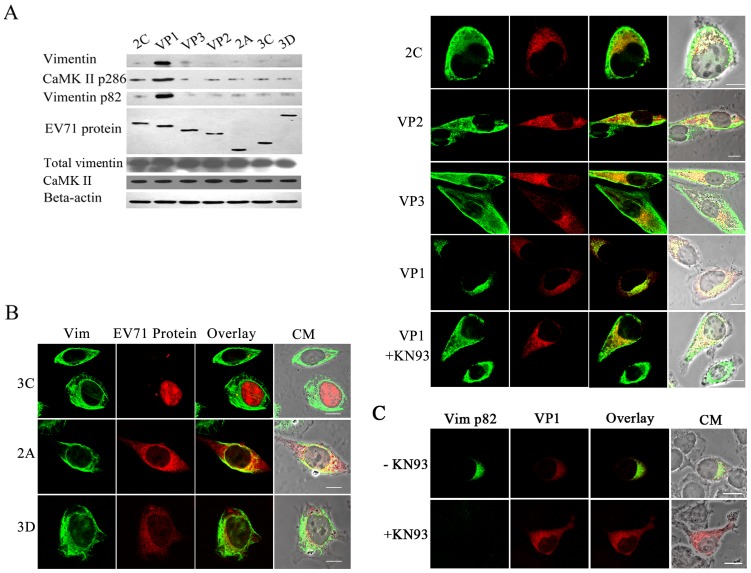
Analysis of EV71 virus proteins involved in CaMK-II activation. (**A**) Western blot analysis of the CaMK-II p286 protein levels in U251 cells expressing various EV71 virus proteins. Cells transfected with each of the recombinant plasmids expressing EV71 2C, VP1, VP2, VP3, 2A, 3C, 3D (as shown on top of the figure) were lysed at 24 h post transfection, and immunoblotted with antibodies to vimentin, CaMK-II, CaMK-II p286, vimentin p82 or Flag tag (EV71 proteins). Total vimentin in cells was also analyzed. Beta-actin was immunoblotted as an internal control. The figure shows a dramatic change in the level of CaMK-II p286 in U251 cells expressing VP1. (**B**) Immunofluorescence analysis of the distribution of vimentin in cells expressing various EV71 proteins at 24 h post transfection. U251 cells were transfected with the recombinant plasmids as described above and stained with antibodies against vimentin (green fluorescence) and Flag (red fluorescence, EV71 proteins). The figure shows the vimentin was disassembled in VP1 expressing cells but no changes in the integrity and distribution of vimentin in celles expressing 2C, VP2, VP3, 2A, 3C, 3D proteins and VP1 expressing cells treated with KN93. Bar: 20 μm. (**C**) Immunofluorescence analysis of the distribution of vimentin p82 (green fluorescence) in VP1 (red fluorescence) expressing cells treated (+KN93) or un-treated (−KN93) with KN93.

To further determine which of the EV71 virus proteins were involved in CaMK-II activation, resulting in vimentin phosphorylation and disassembly, plasmids that expressed either EV71 VP1, VP2, VP3, 2C, 3D, 2A or 3C proteins were transfected separately into U251 cells which were then analyzed using an antibody that specifically recognized threonine 286 of CaM kinase II. The results showed that only the cells expressing VP1 activated CaMK-II as indicated by a dramatic up-regulation of threonine 286 phosphorylation of CaM kinase II (**Fig. 6A**). All the other EV71 proteins-expressing cells showed no change. As expected, the level of ser 82-p vimentin in the VP1 expressing cells, and not others, was significantly up-regulated. Analysis of these cells also showed that EV71 2C, 2A, 3C, 3D, VP3, VP2 expression did not affect the distribution and integrity of the vimentin (**Fig. 6B**). In contrast, in those cells that expressed VP1, the vimentin was disassembled. However, the expressed VP1, accompanied by the disassociation of vimentin, were rearranged to the perinuclear region but did not form aggresomes. In contrast, the vimentin maintained its filamentous structure in VP1 expressing cells when treated with KN93 (**Fig. 6B**). Further evidence showed that the disassembled vimentin in VP1 expressing cells is ser 82 phosphorylated (**Fig. 6C**). When the phosphorylation of vimentin was blocked by KN93, the VP1 was detected throughout the cytoplasm instead of being assembled in the perinuclear region (**Fig. 6C**).

These results indicated that the activation of CaMK-II and the vimentin rearrangement during EV71 infection was mostly attributed to VP1 protein expression, while the formation of aggresomes, the putative virus replication centers, needed the participation of other viral components.

### Involvement of microtubules in the formation of EV71 replication centers

**Figure 7:**
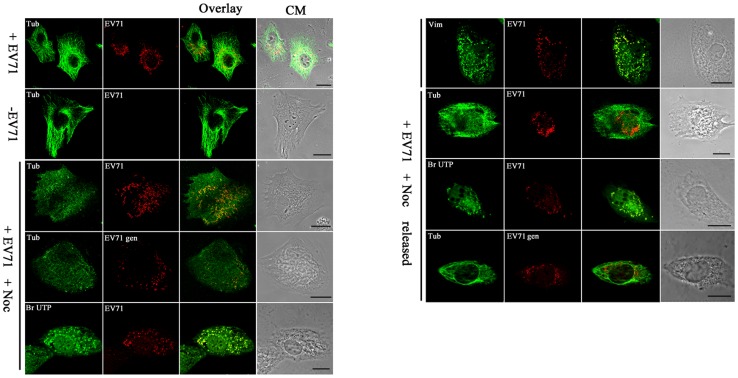
Analysis of the roles of microtubules in the formation of virus replication centers in the perinuclear region. U251 cells were infected or uninfected with EV71 for 22°C in the presence or absence of 3 μM Nocodazol for another 2 h. In one treatment, the Nocodazol added was removed (+N removed) by washing with DMEM and then cultured for another 1 h. Cells in all treatments were then fixed for immunofluorescence studies using antibody raised against EV71 or BrUTP to detect the virus replication centers or rhodamine-conjugated DNA probe complemented with EV71 VP1 gene to detect the EV71 genome. (EV71 gen). Microtubules were labeled with antibody specific for alpha-tubulin (tubulin). In the figure, the panels from top to bottom are: EV71 infected cells (+EV71), uninfected control (−EV71), infected cells treated with Nocodasol (+EV71 + Noc), and infected cells with the Nocodasol removed (+EV71 + Noc released). CM: Cell morphology. Bar: 20 μm.

The rearrangement of vimentin usually involves microtubules [28]. In addition, the perinuclear location of aggresomes implied a role for microtubules and the microtubule organizing center. The distribution of microtubules in cells infected with EV71 was thus studied. U251 cells were stained with antibodies recognizing EV71 and alpha-tubulin at 24 h postinfection. The results showed that, unlike the vimentin filaments, the tubulin maintained its filamentous network reaching the cell surface in infected cells (**Fig. 7**). The ability of the virus to maintain aggresomes in the absence of intact microtubules was tested by adding a microtubule destabilizing drug (Nocodazol) to cells 24 h after infection and then analysed 2 h later. The results showed that after treatment, the microtubules were depolymerized and lost their integrity (**Fig. 7**). It was also found that the aggresomes representing the putative virus replication centers although maintained their integrity, were no longer restricted within the the perinuclear region, but instead dispersed throughout the cytoplasm (**Fig. 7**). However, after removal of the Nocodazol, the aggresomes re-aggregated in the perinuclear region, accompanied by the reorganization of microtubules (**Fig. 7**). The above results suggested that microtubules were not directly involved in EV71 aggresome formation.

## Discussion

The various stages of virus replication and the corresponding processes are closely connected and are interdependent. Therefore, the various components of the virus replication machinery is connected by membranes and other structures to facilitate the synthesis and transport of virus proteins and genomes for assembly and release out of the cells. Despite their inter-connection, each of the components of the virus replication machinery, genome replication, protein synthesis, virus assembly and maturation and transport are compartmentised in specialised structures that known as virus replication centers. These replication centers usually are where the virus components concentrate, thereby increasing the efficiency of the virus replication processes [29]. To date, a variety of unrelated viruses have been reported to induce replication centers in specific areas of the cells, usually at the perinuclear region.

Aggresomes, cytoplasmic inclusions linked to the pathogenesis of many diseases into which aggregated misfolded proteins are sequestered, are thought to immobilize protein aggregates and render them susceptible to proteolysis by proteasomes and/or autophagy [30]. Aggresomes-like structures are also formed in cells infected with various kinds of RNA viruses, where they appear as accumulations of electron dense amorphous materials containing viruses and virus assembly intermediates [7]. In this study, we found that most of the EV71 particles or VP1 proteins and EV71 RNA co-localized approximately with aggresomes-like structures in the perinuclear area. However, the aggresomes induced by EV71 infection seems distinct from that in cells expressing GFP-250. The GFP-250 usually aggregated into a large, single aggresome in perinuclear area. While the aggresomes in EV71 infected cells were numerous and heterogeneous in size. In addition, vimentin filaments in infected cells were rearranged and co-localized with aggresomes. While in GFP-250 expressing cells, vimentin collapse into a ring-like structure around the aggresome. Many studies indicated that the aggresome was participated in virus replication [31]–[33]. Support for this comes from the observation that virus replication was inhibited when the formation of aggresomes were blocked. In our study, when the vimentin rearrangement in EV71 infected cells was blocked, there was a decreasing of EV71 viral production accompanied with the disappearance of aggresomes. Thus, the aggresomes induced by EV71 infection may not be the misfolded viral proteins but the condensed viral components that help virus replication. The aggresomes may provided a scaffold for virus assembly. On the other hand, the aggresomes may increased the local concentration of viral components and thus may increased the productivity of virus assembly.

The formation of virus factories involves a number of complex interactions and signaling events between viral and cellular factors. Mitochondria, cytoplasmic membranes and cytoskeletal components frequently participate in the formation of virus factories, supplying basic needs for carrying key steps in the virus replication cycle [10], [34]. The association of RNA synthesis with intracellular membranes is a typical feature in the replication of some enveloped positive-stranded RNA viruses. For example, togavirus factories are organized around endosomes and lysosomes, while arterivirus factories contain large amounts of double membrane vesicles derived from the endoplasmic reticulum (ER). EV71 infection were shown to induce the formation of auto-phagysomes which might support virus replication but it had now been shown that they were not the virus replication centers. This is in accord with our results which showed that there were no double-membraned autophagosome-like structures in the virus replication areas. Some virus species belonging to the same genus as EV71 had been shown to use membrane structures as replication sites. For example, PV, EV11 and EMCV infections induced heterogeneously-sized vesicles arranged as tightly packed clusters which may represent the virus replication centers. However, the immature or intact EV71 virus-like particles apparently interacted only with filament-like structures and not membrane structures in human astrocyte cells. These filamentous structures could provide a scaffold for attachment of components required for virus replication.

Among the cell skeletons, vimentin and microtubes have been shown to be involved in the replication centers of many virus [35], [36]. For example, vimentin cages have long been known to form around the virus assembly sites of ASFV, Frog virus 3 (FV3) and the pox viruses [31], [37], [38]. Vimentin is the major intermediate filament protein of astrocyte cells and is believed to be responsible for maintaining cell shape, integrity of the cytoplasm and stabilizing cytoskeletal interactions [39]–[41]. It also plays a significant role in supporting and anchoring the nucleus, ER and mitochondria. Its expression was detected in cell of mesenchymal origin and is also present in cells adapted to tissue culture and many transformed cell lines [39]. EV71 infection in astrocyte cells caused a significant rearrangement of vimentin. The rearranged vimentin co-localized well with the EV71 replication centers. This indicated that vimentin was also involved in the formation of EV71 replication centers. Rearrangement of vimentin can be promoted by cellular kinases that phosphorylate the N-terminal domains important for filament assembly. Cdk1 phosphorylates vimentin at Ser 55 from prometaphase to metaphase. Rho-kinase phosphorylates vimentin at Ser 71 specifically at the cleavage furrow from anaphase to the end of mitosis. PKC phosphorylates vimentin Ser 55 and 33. CaMK-II is a multi-subunit enzyme consisting of catalytic, auto regulatory, and subunit assembly domains. Upon activation by calcium and calmodulin, CaMK-II auto phosphorylates threonine 286, leading to full activation of the enzyme. EV71 infection may activate the kinase that specifically phosphorylates vimentin which led to the disassembly of the vimentin filaments and the generation of vimentin aggresomes. Our results showed that EV71 infection activated CaMK-II but not the other protein kinases that are also responsible for vimentin phosphorylation. The rearranged vimentin was serine 82 phosphorylated by CaMK-II and was necessary for the formation of EV71 replication centers since blocking serine 82 phosphorylation by KN93 stopped the generation of aggresomes which represented the virus replication centers. Newly synthesized virus RNA also dispersed into the cytoplasm and not concentrated in the aggresomes after KN93 treatment. ASFV infection also resulted in the activation of CaMK-II and phosphorylation of the N-terminal domain of vimentin. The phosphorylated vimentin concentrated into an “aster” within virus assembly sites located close to the microtubule organizing center [36]. In these virus, vimentin rearranged and formed a scaffold for recruiting virus proteins or genomes necessary for virus replication. In addition, the vimentin might prevent the escape of viral components into the cytoplasm and instead, concentrated structural proteins at the sites of virus assembly [37]. Many studies revealed that vimentin is the organizer for a number of critical proteins involved in attachment, migration and cell signaling [40], [42]. The highly dynamic and complex phosphorylation of vimentin could be the likely regulatory mechanism for these functions. Whatever function vimentin has, its rearrangement by EV71 infection definitely influenced its normal cellular functions.

Astrocyte cells are the most abundant cell type in the human brain [43]. They perform many important functions, including biochemical support of endothelial cells that form the blood brain barrier, provision of nutrients to the nervous tissue, maintenance of extracellular ion balance and providing a role in the repair and scarring process of the brain and spinal cord following traumatic injuries [44]. Many studies showed that astrocytes propagate intercellular Ca^2+^ waves over long distances in response to stimulation and, similar to neurons, release transmitters in a Ca^2+^ dependent manner [45], [46]. CaMK-II is a serine/threonine-specific protein kinase that is regulated by the Ca^2+^/calmodulin complex, and is believed to be involved in many signaling cascades as well as being an important mediator of learning and memory [47], [48]. Mis-regulation of CaMK-II was linked to Alzheimer's disease and Angelman's syndrome. In this study, we found that EV71 infection activated CaMK-II. However, how CaMK -II was activated is still unknown. Perhaps EV71 infection led to an increase in Ca^2+^ concentration in cells, resulting in CaMK-II activation or, EV71 proteins mimicked Ca^2+^/calmodulin complex and activated CaMK-II.

Several studies showed that cell apoptosis caused by virus infection, combining with tissue damage to induce toxic inflammatory cytokines and cellular immunity, as being the possible cause of pathogenesis in the CNS. Based on this study, we could hypothesize that some EV71 associated syndromes may be attributed to the vimentin rearrangement and mis-regulation of CaMK-II during infection. For example, the rearrangement of vimentin could affect the normal positioning of the organelles in the cytosol and weaken cell-cell attachment, and thus destroying the transmission of neural signals. In addition, irregular distribution of vimentin in astrocyte cells could affect their biochemical support of endothelial cells, thus destroying the blood brain barrier, and leading to encephalitis. Further, the mis-regulation of CaMK-II could affect the ion balance of CNS and thus impacting nerve action. However, further research is required to ascertain whether CNS syndrome during EV71 infection is related to vimentin rearrangement and CaMK-II activation.

**Figure 8:**
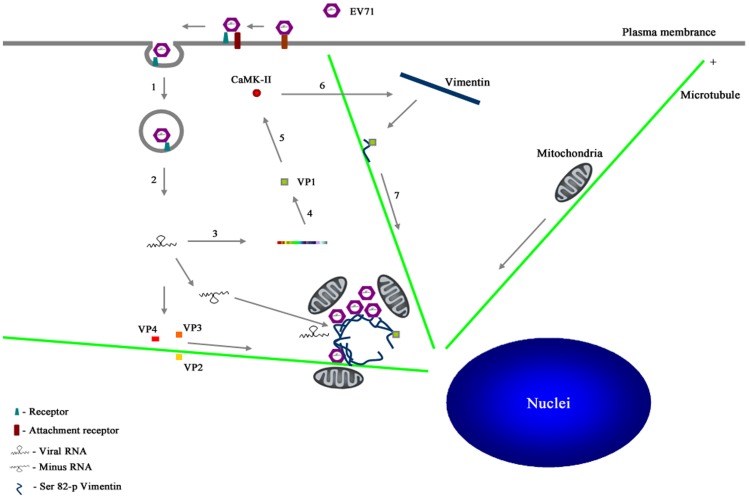
A model for the formation of EV71 replication centers in U251 cells. 1. EV71 virions enter the cell by receptor-mediated endocytosis. 2. The virus is uncoated and the viral genome is released to the cytoplasm. 3. Genomic RNA is first translated to produce the viral polyprotein. 3. The polyprotein is then processed to produce the various precursors and processed proteins that are needed for EV71 replication. 4. The synthesized virus VP1 protein mediates the phosphorylation of CaMK-II at Thr286 and resulting in CaMK-II activation. 5. The activated CaMK-II phosphorylates vimentin filaments at Ser82 and causes vimentin filaments to disassemble. 6. The VP1 combines with the disassembled vimentin and are transported to the perinuclear region and initiate replication center formation. The replication centers provide a scaffold for viral genome synthesis. Newly synthesized RNA enters either the translation-replication cycle or the virus particle assembly step. Mitochondria are recruited to near the replication centers to provide energy that is needed for viral genome synthesis. During infection, other virus structural proteins are carried by hypothetical virus carrier proteins along microtubules to the replication centers to facilitate further assembly.

In conclusion, this study delivers important findings on the roles of vimentin filaments in EV71 replication. Based on this study, we propose a model (**Fig. 8**) showing that EV71 infection led to the activation of CaMK-II by VP1, resulting in the phosphorylation of serine 82 of the vimentin filaments. Presumably, this phosphorylation reduced the interaction between the N-terminal domains of the vimentin filaments and facilitated their disassembly. The disassembled vimentin then combined with EV71 viral components and microtubules to form the virus replication centers in the perinuclear regions. At the same time, the virus replication process recruited mitochondria which facilitated virus replication. Thus, the present study provides insight into the EV71 replication process in human astrocyte cells, and helps us to understand the pathogenesis of CNS syndromes caused by EV71 infection.

## Supporting Information

Figure S1 **A comparison of vimentin distribution in cells expressing GFP-250 (top panels, green fluorescence) and cells infected with EV71 (bottom panels).** U251 cells were transfected with pcDNA-GFP-250. 24 hours post transfection, cells were fixed and stained with antibodies to vimentin (Vim, red fluorescence) and subjected to confocal microscopy analysis. Some U251 cells were infected with EV71 for 24 hours. Cells were then fixed and stained with antibodies to vimentin (Vim, red fluorescence) and EV71 (EV71, green fluorescence). Bar: 20 μm.
(DOC)

Figure S2 **Measurement of virus titers in the supernatants of cells treated (+KN93) or untreated (−KN93) with KN93.** Uninfected U251 cells as controls (U251 – EV71). Virus titration was performed at 0, 12 and 24 h postinfection. The data show the mean virus titers ± SD from three independent experiments. Asterisks indicate significant differences at p<0.05 compared to control.
(DOC)

## References

[1] Chang LY, Lin TY, Hsu KH, Huang YC, Lin KL, et al (1999) Clinical features and risk factors of pulmonary oedema after enterovirus-71-related hand, foot, and mouth disease. Lancet 354: 1682–1686. 10568570 10.1016/S0140-6736(99)04434-7

[2] Ooi MH, Wong SC, Lewthwaite P, Cardosa MJ, Solomon T (2010) Clinical features, diagnosis, and management of enterovirus 71. Lancet Neuro l 9: 1097–1105. 10.1016/S1474-4422(10)70209-X 20965438

[3] Huang CC, Liu CC, Chang YC, Chen CY, Wang SY, et al (1999) Neurologic complications in children with enterovirus 71 infection. N Engl J Med 341: 936–942. 10498488 10.1056/NEJM199909233411302

[4] Lu J, Yi L, Zhao J, Yu J, Chen Y, et al (2012) Enterovirus 71 disrupts interferon signaling by reducing the level of interferon receptor 1. J Virol 86: 3767–3776. 22258259 10.1128/JVI.06687-11 PMC3302529

[5] Lin YW, Chang KC, Kao CM, Chang SP, Tung YY, et al (2009) Lymphocyte and antibody responses reduce enterovirus 71 lethality in mice by decreasing tissue viral loads. J Virol 83: 6477–6483. 19386699 10.1128/JVI.00434-09 PMC2698549

[6] Chang LY, Huang LM, Gau SSF, Wu YY, Hsia HS, et al (2007) Neurodevelopment and cognition in children after enterovirus 71 infection. N Engl J Med 356: 1226–1234. 17377160 10.1056/NEJMoa065954

[7] Wileman T (2006) Aggresomes and autophagy generate sites for virus replication. Science 312: 875–878. 16690857 10.1126/science.1126766

[8] Wong J, Zhang JC, Si XN, Gao G, Mao I, et al (2008) Autophagosome supports coxsackievirus B3 replication in host cells. J Virol 82: 9143–9153. 18596087 10.1128/JVI.00641-08 PMC2546883

[9] Huang SC, Chang CL, Wang PS, Tsai Y, Liu HS (2009) Enterovirus 71-induced autophagy detected in vitro and in vivo promotes viral replication. J Med Virol 81: 1241–1252. 19475621 10.1002/jmv.21502 PMC7166624

[10] Novoa RR, Calderita G, Arranz R, Fontana J, Harald G, et al (2005) Virus factories: associations of cell organelles for viral replication and morphogenesis. Biol Cell 97: 147–172. 15656780 10.1042/BC20040058 PMC7161905

[11] Sharpe AH, Chen LB, Fields BN (1982) The interaction of mammalian reoviruses with the cytoskeleton of monkey kidney CV-1 cells. Virology 120: 399–411. 7201720 10.1016/0042-6822(82)90040-x

[12] Chou YH, Rosevear E, Goldman RD (1989) Phosphorylation and disassembly of intermediate filaments in mitotic cells. Proc Natl Acad Sci U S A 86: 1885–1889. 2648386 10.1073/pnas.86.6.1885 PMC286809

[13] Inagaki M, Nishi Y, Nishizawa K, Matsuyama M, Sato C (1987) Site-specific phosphorylation induces disassembly of vimentin filaments in vitro. Nature 328: 649–652. 3039376 10.1038/328649a0

[14] Tsujimura K, Ogawara M, Takeuchi Y, Imajoh-Ohmi S, Ha MH, et al (1994) Visualization and function of vimentin phosphorylation by cdc2 kinase during mitosis. J Biol Chem 269: 31097–31106. 7983050

[15] Minetaro A, Hiroyuki S, Noriyo N, Yasushi A, Yuriko S, et al (2005) Temperature-sensitive mutants of enterovirus 71 show attenuation in cynomolgus monkeys. J Gen Virol 86: 1391–1401. 15831951 10.1099/vir.0.80784-0

[16] Jan P, Elizabeth HM (1968) Long term culture of normal and neoplastic human glia. Acta Pathol Microbiol Scand 74: 465–486. 4313504 10.1111/j.1699-0463.1968.tb03502.x

[17] Robert MM, John M, Jerry ZF, Ernest CA, Murray BG (1969) Cultivation in vitro of cells derived from a human rhabdomyosarcoma. Cancer 24: 520–526. 4241949 10.1002/1097-0142(196909)24:3<520::aid-cncr2820240313>3.0.co;2-m

[18] Kim H, Nakamura F, Lee W, Shifrin YL, Arora P, et al (2009) Filamin A is required for vimentin-mediated cell adhesion and spreading. Am J Physiol Cell Physiol 298: C221–C236. 19776392 10.1152/ajpcell.00323.2009 PMC4380480

[19] Oker-Blom C, Kalkkinen N, Kääriäinen L, Pettresson RF (1983) Rubella virus contains one capsid protein and three envelope glycoproteins, E1, E2a, and E2b. J Virol 46: 964–973. 6854740 10.1128/jvi.46.3.964-973.1983 PMC256571

[20] Hooghe-Peters EL, Rentier B, Dubois-Dalcq M (1979) Electron microscopic study of measles virus infection: unusual antibody-triggered redistribution of antigens on giant cells. J Virol 29: 666–676. 107326 10.1128/jvi.29.2.666-676.1979 PMC353199

[21] Cong H, Jiang Y, Po T (2011) Identification of the Myelin Oligodendrocyte Glycoprotein as a Cellular Receptor for Rubella Virus. J Virol 85: 11038–11047. 21880773 10.1128/JVI.05398-11 PMC3194935

[22] Vyboh K, Ajamian L, Mouland AJ (2012) Detection of viral RNA by fluorescence in situ hybridization (FISH). J Vis Exp 63: e4002. 10.3791/4002 PMC3466955 22588480

[23] García-Mata R, Bebök Z, Sorscher EJ, Sztul ES (1999) Characterization and dynamics of aggresome formation by a cytosolic GFP-chimera. J Cell Biol 146: 1239–1254. 10491388 10.1083/jcb.146.6.1239 PMC2156127

[24] Yasui Y, Gotu H, Matsui S, Manser E, Lim L, et al (2001) Protein kinases required for segregation of vimentin filaments in mitotic process. Oncogene 20: 2868–2876. 11420699 10.1038/sj.onc.1204407

[25] Goto H, Kosako H, Tanabe K, Yanagida M, Sakurai M, et al (1998) Phosphorylation of vimentin by Rho-associated kinase at a unique amino-terminal site that is specifically phosphorylated during cytokinesis. J Biol Chem 273: 11728–11736. 9565595 10.1074/jbc.273.19.11728

[26] Yamaguchi T, Goto H, Yokoyama T, Silljé H, Hanisch A, et al (2005) Phosphorylation by Cdk1 induces Plk1-mediated vimentin phosphorylation during mitosis. J Cell Biol 171: 431–436. 16260496 10.1083/jcb.200504091 PMC2171270

[27] Yang E, Schulman H (1999) Structural examination of autoregulation of multifunctional calcium/calmodulin-dependent protein kinase II. J Biol Chem 274: 26199–26208. 10473573 10.1074/jbc.274.37.26199

[28] Hanson LK, Slater JS, Cavanaugh VJ, Newcomb WW, Bolin LL, et al (2009) Murine cytomegalovirus capsid assembly is dependent on US22 family gene M140 in infected macrophages. J Virol 83: 7449–7456. 19458005 10.1128/JVI.00325-09 PMC2708628

[29] Wileman T (2007) Aggresomes and pericentriolar sites of virus assembly: cellular defense or viral design? Annu Rev Microbiol 61: 149–167. 17896875 10.1146/annurev.micro.57.030502.090836

[30] Rubinsztein DC (2006) The roles of intracellular protein-degradation pathways in neurodegeneration. Nature 443: 780–786. 17051204 10.1038/nature05291

[31] Heath CM, Windsor M, Wileman T (2001) Aggresomes resemble sites specialized for virus assembly. J Cell Biol 153: 449–455. 11331297 10.1083/jcb.153.3.449 PMC2190574

[32] Leão Ferreira RL, Moussatche N, Neto VM (1994) Rearrangement of intermediate filament network of BHK-21 cells infected with vaccinia virus. Arch Virol. 138: 273–285. 10.1007/BF01379131 7998834

[33] Murti KG, Goorha R (1983) Interaction of frog virus-3 with the cytoskeleton. I. Altered organization of microtubules, intermediate filaments, and microfilaments. J Cell Biol 96: 1248–1257. 6341377 10.1083/jcb.96.5.1248 PMC2112641

[34] Netherton C, Moffat K, Brooks E, Wileman T (2007) A guide to viral inclusions, membrane rearrangements, factories, and viroplasm produced during virus replication. Adv Virus Res 70: 101–182. 17765705 10.1016/S0065-3527(07)70004-0 PMC7112299

[35] Dohner K, Sodeik B (2005) The role of the cytoskeleton during viral infection. Curr Top Microbiol Immunol 285: 67–108. 15609501 10.1007/3-540-26764-6_3

[36] Radtke K, Dohner K, Sodeik B (2006) Viral interactions with the cytoskeleton: a hitchhiker's guide to the cell. Cell Microbiol 8: 387–400. 16469052 10.1111/j.1462-5822.2005.00679.x

[37] Stefanovic S, Windsor M, Nagata KI, Inagaki M, Wileman T (2005) Vimentin rearrangement during African swine fever virus infection involves retrograde transport along microtubules and phosphorylation of vimentin by calcium calmodulin kinase II. J Virol 79: 11766–11775. 16140754 10.1128/JVI.79.18.11766-11775.2005 PMC1212593

[38] Smith GA, Enquist LW (2002) Break ins and break outs: viral interactions with the cytoskeleton of Mammalian cells. Annu Rev Cell Dev Biol 18: 135–161. 12142276 10.1146/annurev.cellbio.18.012502.105920

[39] Eriksson JE, Dechat T, Grin B, Helfand B, Mendez M, et al (2009) Introducing intermediate filaments: from discovery to disease. J Clin Invest 119: 1763–1771. 19587451 10.1172/JCI38339 PMC2701876

[40] Goldman RD, Khuon S, Chou YH, Opal P, Steinert PM (1996) The function of intermediate filaments in cell shape and cytoskeletal integrity. J Cell Biol 134: 971–983. 8769421 10.1083/jcb.134.4.971 PMC2120965

[41] Azumi N, Battifora H (1987) The distribution of vimentin and keratin in epithelial and nonepithelial neoplasms. A comprehensive immunohistochemical study on formalin- and alcohol-fixed tumors. Am J Clin Pathol 88: 286–296. 2443000 10.1093/ajcp/88.3.286

[42] Ivaska J, Pallari HM, Nevo J, Eriksson JE (2007) Novel functions of vimentin in cell adhesion, migration, and signaling. Exp Cell Res 313: 2050–2062. 17512929 10.1016/j.yexcr.2007.03.040

[43] Volterra A, Meldolesi J (2005) Astrocytes, from brain glue to communication elements: the revolution continues. Nat Rev Neurosci 6: 626–640. 16025096 10.1038/nrn1722

[44] Pascual O, Casper KB, Kubera C, Zhang J, Revilla-Sanchez R, et al (2005) Astrocytic purinergic signaling coordinates synaptic networks. Science 310: 113–116. 16210541 10.1126/science.1116916

[45] Santello M, Volterra A (2009) Synaptic modulation by astrocytes via Ca^2+^-dependent glutamate release. Neuroscience 158: 253–259. 18455880 10.1016/j.neuroscience.2008.03.039

[46] Piet R, Vargová L, Syková E, Poulain DA, Oliet SH (2004) Physiological contribution of the astrocytic environment of neurons to intersynaptic crosstalk. Proc Natl Acad Sci U S A 101: 2151–2155. 14766975 10.1073/pnas.0308408100 PMC357067

[47] Giese KP, Fedorov NB, Filipkowski RK, Silva AJ (1998) Autophosphorylation at Thr286 of the alpha calcium-calmodulin kinase II in LTP and learning. Science 279: 870–873. 9452388 10.1126/science.279.5352.870

[48] Irvine EE, von Hertzen LS, Plattner F, Giese KP (2006) alphaCaMK-II auto-phosphorylation: a fast track to memory. Trends Neurosci 29: 459–465. 16806507 10.1016/j.tins.2006.06.009

