# Supplementary figures and images for: Enterovirus 71 VP1 Activates Calmodulin-Dependent Protein Kinase II and Results in the Rearrangement of Vimentin in Human Astrocyte Cells
Source: PLoS One. 2013 Sep 20;8(9):e73900. doi: 10.1371/journal.pone.0073900 (PMC3779202; doi:10.1371/journal.pone.0073900)

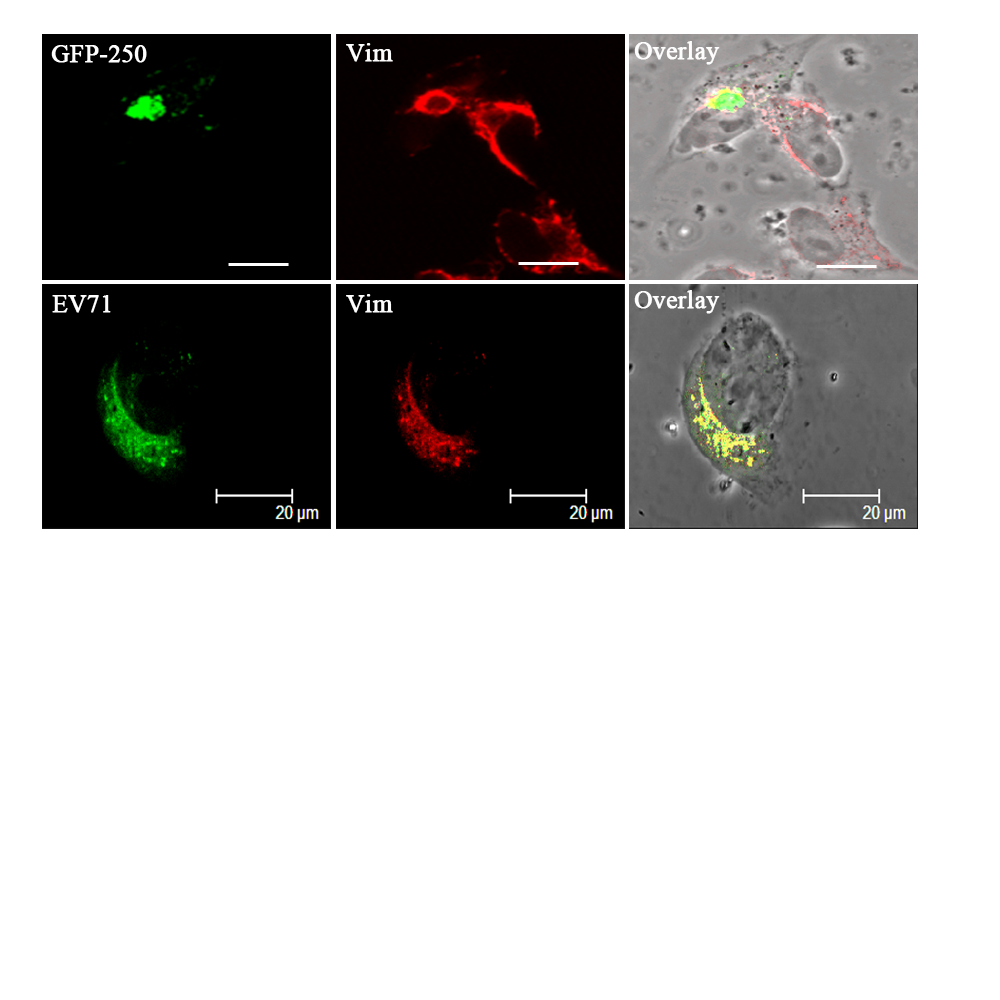

Supplement: Figure S1 — A comparison of vimentin distribution in cells expressing GFP-250 (top panels, green fluorescence) and cells infected with EV71 (bottom panels). U251 cells were transfected with pcDNA-GFP-250. 24 hours post transfection, cells were fixed and stained with antibodies to vimentin (Vim, red fluorescence) and subjected to confocal microscopy analysis. Some U251 cells were infected with EV71 for 24 hours. Cells were then fixed and stained with antibodies to vimentin (Vim, red fluorescence) and EV71 (EV71, green fluorescence). Bar: 20 μm. (DOC) [file pone.0073900.s001.doc]

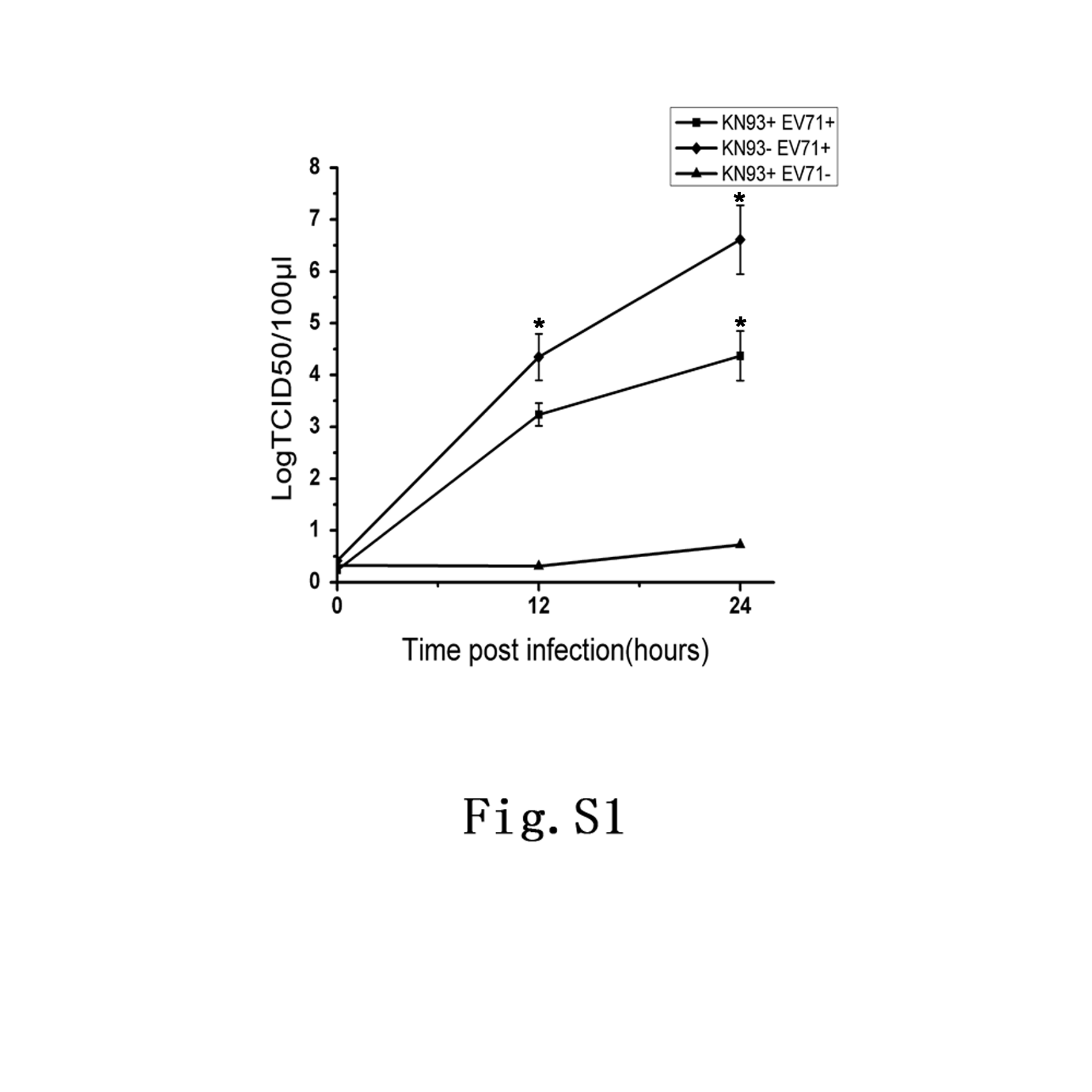

Supplement: Figure S2 — Measurement of virus titers in the supernatants of cells treated (+KN93) or untreated (−KN93) with KN93. Uninfected U251 cells as controls (U251 – EV71). Virus titration was performed at 0, 12 and 24 h postinfection. The data show the mean virus titers ± SD from three independent experiments. Asterisks indicate significant differences at p<0.05 compared to control. (DOC) [file pone.0073900.s002.doc]
